# Risk Scores for Stratifying Hepatocellular Carcinoma and Optimizing Surveillance Strategies

**DOI:** 10.3390/cancers18010158

**Published:** 2026-01-02

**Authors:** Yu-Ping Chang, Yun-Chu Chen, Chen-Hua Liu

**Affiliations:** 1 Department of Internal Medicine, National Taiwan University Biomedical Park Hospital, Hsinchu 302058, Taiwan; melsachangchang@gmail.com; 2 Department of Internal Medicine, National Taiwan University Hospital, Taipei 100229, Taiwan; ycc103311151@gmail.com; 3 Hepatitis Research Center, National Taiwan University Hospital, Taipei 100229, Taiwan; 4 Department of Internal Medicine, National Taiwan University Hospital, Yun-Lin Branch, Douliou 640203, Taiwan

**Keywords:** risk scores, risk stratification, hepatocellular carcinoma surveillance

## Abstract

**Background**: Hepatocellular carcinoma (HCC) is a major global health burden, with poor outcomes largely due to diagnosis at an advanced stage and the limited performance of current surveillance tools. Ultrasound with alpha fetoprotein (AFP) provides insufficient sensitivity for early-stage detection, highlighting the need to better identify the at-risk population. **Focus of the review**: Many HCC risk scores have been proposed; however, some depend on specialized laboratory data that are not widely available. This review summarizes risk scores that show reliable discrimination and rely on demographic, clinical, or molecular information that can be readily obtained in routine care. **Conclusions**: Advances in HCC risk scores support the move toward surveillance approaches based on individual risk. These tools can improve risk stratification, increase the likelihood of early detection, and potentially support better outcomes for people who belong to the at-risk population for HCC.

## Simple Summary

This review highlights the limitations of current hepatocellular carcinoma (HCC) surveillance and emphasizes the need for improved identification of individuals at risk. It summarizes validated HCC risk scores that rely on readily available demographic, clinical, or molecular parameters, rather than specialized or inaccessible tests.

## 1. Introduction

### 1.1. Epidemiology and Global Burden of HCC

The global incidence of liver cancer is projected to nearly double between 2022 and 2050, reaching an estimated 1.5 million new cases by 2050 [1]. Hepatocellular carcinoma (HCC) is the most common type of liver cancer, which accounts for approximately 75–86% of all cases [2]. It is the sixth most common malignancy, and the third leading cause of cancer-related mortality worldwide [3]. HCC continues to impose a tremendous global health burden, accounting for an estimated 12.9 million disability-adjusted life years (DALYs) in 2021, one of the highest among all cancers, largely driven by late-stage diagnosis and correspondingly high mortality. The absolute number of DALYs continues to rise due to population growth and aging [4]. Regionally, East Asia and sub-Saharan Africa continue to bear the highest DALY burden, largely due to endemic hepatitis B and C, while Western Europe and North America have seen rising DALYs related to alcohol and metabolic dysfunction-associated steatotic liver disease (MASLD) [5]. Globally, the overall 5-year survival rate for HCC remains poor, at approximately 20%. Outcomes vary markedly by tumor stage at diagnosis, treatment type and time period of diagnosis [6,7]. Recent real-world data analyzed by Mignote et al. underscore the profound impact of stage migration and treatment accessibility on survival. Notably, patients diagnosed in the more recent period (2013–2019) achieved better overall survival compared with those diagnosed in the earlier period (2006–2012) (*p* < 0.001), reflecting advances in early detection and broader access to curative treatments [8]. Survival gains have been most pronounced in patients detected at early stages and treated with curative interventions, reflected in 5-year survival rates of 70–75% [9], whereas those diagnosed at intermediate or advanced stages continue to have a poor prognosis due to reliance on palliative locoregional or systemic therapies [10]. Although improvements in overall survival were found over the past decade, the majority of patients are still diagnosed at advanced stages where curative treatments are no longer feasible [7]. These findings underscore the substantial public health and economic impact of HCC worldwide.

### 1.2. Shifting Etiologies of HCC: From Viral Hepatitis to Metabolic Dysfunction-Associated Steatotic Liver Disease (MASLD) and Alcohol-Related Liver Disease (ALD)

Worldwide, the most common etiologies of HCC are chronic hepatitis B virus (HBV) infection (44%), chronic hepatitis C virus (HCV) infection (21%), alcohol-related liver disease (ALD, 12–15%), and metabolic dysfunction-associated steatotic liver disease (MASLD, estimated 10–20%) [11]. These proportions vary substantially by geographic region and sex. HBV remains the predominant cause in Asia and Africa, whereas HCV, ALD, and MASLD contribute more prominently to HCC in Western countries [11,12,13,14]. Sex-specific differences are also evident: ALD- and HBV-related HCC exhibit higher age-standardized rates in men, while HCV- and MASLD-related HCC show relatively higher rates in women [15]. Importantly, the etiological landscape of HCC is undergoing a marked shift. Recent trends indicate a global decline in HBV- and HCV-related HCC incidence, driven primarily by widespread neonatal HBV vaccination and the availability of effective antiviral treatments for both HBV and HCV [16]. This decline is most evident in regions with robust public health interventions—such as East Asia for HBV and the United States and Europe for HCV—where direct-acting antivirals have led to a rapid reduction in HCV-related HCC since 2015 [16,17]. Concurrently, the prevalence of MASLD and its progressive form, metabolic dysfunction-associated steatohepatitis (MASH), continues to rise globally, driven by increasing rates of obesity, diabetes, and metabolic syndrome [18,19]. In the United States, MASLD has become the leading cause of HCC among women and is rapidly increasing among all populations, while HCV-related HCC is declining [14]. In Asia, although viral hepatitis remains the predominant etiology, the burden of MASLD and alcohol-related HCC is also increasing, particularly in younger populations [20]. Recent analyses show that the proportion of alcohol-related HCC is projected to increase from 18.8% in 2022 to 21.1% in 2050; and MASLD-related HCC is projected to increase from 8.0% in 2022 to 10.8% in 2050 [21]. As MASLD affects more than 30% of adults, the at-risk population now extends far beyond traditional liver cancer risk groups—a shift that requires urgent prioritization [22].

### 1.3. Current Surveillance Strategies and Cost-Effectiveness Considerations

Surveillance strategies for HCC aim to detect tumors at an early stage, facilitate curative-intent treatment, and are simultaneously guided by cost-effectiveness considerations informed by decision-analytic frameworks such as Markov models. These models incorporate key parameters—including HCC incidence, the sensitivity and specificity of surveillance modalities, downstream diagnostic and treatment costs, quality-adjusted life years (QALYs) gained, and willingness-to-pay thresholds (typically US$50,000–100,000 per QALY in the United States)—to determine when surveillance provides net clinical and economic benefit [23]. Surveillance is considered cost-effective when the incremental cost per QALY gained falls below the accepted threshold and when the annual incidence of HCC in the target population exceeds the modeled cutoff: generally ≥1% for patients with cirrhosis, and approximately 0.2% for high-risk individuals with chronic hepatitis B [23,24,25].

These principles form the basis of current recommendations from major liver societies, including the American Association for the Study of Liver Diseases (AASLD), the European Association for the Study of the Liver (EASL), and the Asian Pacific Association for the Study of the Liver (APASL), unless they have a relatively high risk of death from non-HCC causes, or could not receive a curative-intent treatment for HCC [2,26,27]. The annual incidence of HCC in patients with cirrhosis of any cause is between 1% and 4%. The highest rates are observed in those with hepatitis C virus-related cirrhosis (up to 3.3 per 100 person-years), followed by hepatitis B virus-related cirrhosis (approximately 3.2 per 100 person-years), then alcoholic liver disease-related cirrhosis (0.86–2.06 per 100 person-years), and the lowest rates are seen in MASLD or MASH-related cirrhosis (0.90–1.35 per 100 person-years) [9,28,29,30]. Accordingly, all patients with Child–Pugh A or B cirrhosis, regardless of etiology, should undergo routine surveillance. Patients with Child–Pugh C cirrhosis are eligible for surveillance only if they are transplant candidates [2,26,27]. For non-cirrhotic chronic hepatitis B, the cost-effectiveness threshold is lower. Surveillance is recommended for individuals whose annual HCC incidence exceeds 0.2%, including men over 40 and women over 50 from endemic regions, persons from Africa who warrant earlier screening (beginning in the third decade of life), those with a family history of HCC, and patients with a PAGE-B score ≥ 10 (a validated HBV risk model based on age, sex, and platelet count) [31]. In contrast, individuals with insufficient risk to justify routine surveillance, such as non-cirrhotic MASLD or HCV patients with advanced fibrosis (F3), typically have an annual HCC incidence below 0.2%, and therefore require further risk stratification using validated prediction models to determine whether surveillance should be initiated [2,26,27,32].

The most widely used surveillance tool for HCC in the at-risk population is biannual ultrasonography, with or without alpha-fetoprotein (AFP) testing, owing to its noninvasive nature and robust evidence supporting its cost-effectiveness [2,23,26,27]. For HCC of all tumor stages, ultrasonography alone demonstrates a sensitivity of 72–84% and a specificity of 91–96%. When combined with alpha-fetoprotein (AFP) testing at a cutoff of 20 ng/mL, sensitivity increases to approximately 96%, with specificity around 85%. For early-stage HCC, however, performance is substantially lower: ultrasonography alone yields a sensitivity of only 47–53% (specificity 91–96%), and adding AFP increases sensitivity modestly to about 63% (specificity 85–90%), implying that even with combined testing, nearly one-third of early HCC cases remain undetected [9,33,34,35,36,37,38]. Notably, lowering AFP cutoff values from the traditional 20 ng/mL to approximately 10–14 ng/mL improves sensitivity for HCC detection while maintaining comparable specificity, with the greatest benefit observed in patients with non-viral liver disease such as MASLD and alcohol-associated liver disease(ALD) [39,40,41].

### 1.4. Challenges and Gaps in Current HCC Surveillance

Current HCC surveillance strategies face substantial limitations and unmet clinical needs. Barriers to surveillance include limited disease awareness, provider knowledge gaps, disparities in access to subspecialty care, and difficulty identifying at-risk patients [42,43]. Surveillance uptake is poor worldwide; multiple meta-analyses and large cohort studies confirm that less than 25% of at-risk individuals (primarily those with cirrhosis or chronic hepatitis B) receive surveillance every 6 months, and often much lower for strict adherence to biannual imaging [44,45]. Up to 40% of MASLD-related HCC occurs in individuals without cirrhosis; consequently, many at-risk patients fall outside traditional cirrhosis-based surveillance criteria, underscoring the urgent need for more precise risk stratification [46].

Ultrasonography, the cornerstone of HCC surveillance, is highly operator-dependent and has reduced sensitivity in patients with obesity and MASLD, leading to missed early-stage tumors and delayed diagnosis [47]. These challenges are amplified by shifting disease epidemiology from viral hepatitis to MASLD. To address the limitations of ultrasonography, recent studies demonstrate that abbreviated MRI (AMRI) and non-contrast MRI offer substantially higher sensitivity and specificity for early-stage HCC than ultrasound [48,49]. Consistent with this, AASLD recommends documenting ultrasound adequacy using the Liver Imaging Reporting and Data System (LI-RADS) visualization score and pursuing alternative imaging when examinations are severely limited [2].

Serum biomarkers beyond AFP, such as protein induced by vitamin K absence II (PIVKA-II), also known as des-gamma-carboxy prothrombin (DCP), are particularly valuable for identifying HCC in AFP-negative patients and demonstrate comparable or slightly superior sensitivity to AFP for detecting early-stage disease [50,51]. Similarly, Alpha-fetoprotein–L3 (AFP–L3) is a glycoform of alpha-fetoprotein (AFP) that is specifically produced by malignant hepatocytes. Measurement of AFP–L3%—the proportion of AFP–L3 to total AFP—improves the specificity of HCC diagnosis, but demonstrates moderate to low sensitivity for early HCC detection, making it most useful for confirming HCC in patients with elevated AFP, rather than as a standalone screening tool [52]. Neither PIVKA–II nor AFP–L3% is recommended for routine surveillance because of their limited sensitivity when used alone [53].

### 1.5. Novel Biomarkers and Microenvironment-Based Prediction Tools

Novel biomarkers that reflect malignant transformation and aberrant glycosylation processes rather than hepatocellular injury or fibrosis are under investigation. These markers provide a more direct indication of oncogenic activity and include Golgi protein 73 (GP73) [54,55], glypican-3 (GPC3) [56,57], osteopontin [58,59], circulating tumor DNA (ctDNA) [60], and cell-free DNA (cfDNA) [61,62]. GP73, GPC3, and osteopontin demonstrate slightly better or comparable sensitivity to AFP for HCC diagnosis; however, none consistently outperforms ultrasound or AFP alone. Their primary value lies in providing incremental sensitivity when used in combination with AFP rather than as standalone surveillance tools [55,63,64]. Liquid biopsy using ctDNA and cfDNA and methylation-based panels is increasingly recognized for its clinical value in risk stratification, early detection, prognosis assessment, and disease monitoring [65,66]. Recent multicenter cohort studies and prospective validations have demonstrated that methylation-based ctDNA assays can achieve sensitivities and specificities exceeding 85% for HCC diagnosis, and outperform mutation-based ctDNA approaches [67]. Urine-based cfDNA methylation panels and combined blood-based multi-marker models (e.g., HCCtect, MCP technology) have shown incremental sensitivity over AFP alone, particularly for early-stage and AFP-negative HCC, with area under the ROC curve (AUC) values frequently above 0.90 [62,68]. Beyond circulating biomarkers, recent studies have highlighted the potential of microenvironment-based prediction tools that integrate spatial, cellular, and molecular features of the hepatic microenvironment. For example, advanced models incorporating tissue-level architecture and cellular interactions have demonstrated the ability to identify high-risk phenotypes and refine prognostic stratification [69]. Although these approaches remain largely investigational, they may eventually complement existing clinical risk scores by providing mechanistic insights into hepatocarcinogenesis and enabling more individualized surveillance strategies. Despite these advances, the AASLD advises against the diagnosis of hepatocellular carcinoma based on biomarkers or liquid biopsy until further validation in Phase III–IV biomarker cohort studies [2].

## 2. Evolution and Validation of Risk Scores for HCC Surveillance

Identifying the at-risk population is the foundational step for implementing precision surveillance, as the risk of HCC among patients with chronic liver disease is highly heterogeneous. This variability necessitates more refined methods of risk estimation beyond traditional cirrhosis-based criteria. The widely used Fibrosis-4 (FIB-4) index, originally developed to assess liver fibrosis in patients co-infected with HCV and human immunodeficiency virus (HIV), relies solely on age, aspartate aminotransferase (AST), alanine aminotransferase (ALT), and platelet count. However, it does not incorporate important HCC risk factors, including sex, hepatitis B virus DNA (HBV DNA) levels, diabetes, and treatment status. Its discriminative ability for HCC is moderate (AUC: 0.70–0.78) [70]. To address this limitation, new risk scores were developed, including etiology-specific models and mixed-etiology models, which incorporate additional clinical and virologic variables that independently predict HCC risk [70,71,72,73]. Ongoing clinical trials and real-world studies continue to evaluate their performance and clinical utility [74].

### 2.1. Etiology-Specific Risk Scores (Table 1)

#### 2.1.1. Hepatitis B Virus (HBV)

The PAGE-B score, developed in 2016 for European patients with chronic HBV on antiviral treatment, uses age, sex, and platelet count to stratify HCC risk. It reliably identifies low-risk patients (score ≤ 9) with a near-zero incidence of HCC over 5 years and demonstrates a high negative predictive value in Asian and Caucasian (Europe, Australia, United States) cohorts, with AUC up to 0.82 in validation studies [31,75,76,77]. The modified PAGE-B (mPAGE-B), developed in 2018, by adding albumin to the original score, has shown improved discrimination for HCC risk prediction across both Asian and non-Asian populations receiving antiviral treatments [75,78,79]. Yip et al. included more than 32,000 patients treated with nucleos(t)ide analogs, individuals classified as low risk (29.3% of the cohort) by either PAGE-B or mPAGE-B demonstrated an exceptionally low annual HCC incidence of <0.2%. When both criteria were met (PAGE-B < 10 and mPAGE-B < 9), the 5-year negative predictive value reached 99.7%, strongly supporting the utility of these scores for identifying patients who may be exempt from intensive HCC surveillance over the next 5 years [75].

The Risk Estimation for Hepatocellular Carcinoma in Chronic Hepatitis B (REACH-B) score, developed in 2011 from the untreated, non-cirrhotic Taiwanese REVEAL-HBV cohort, incorporates age, sex, ALT, hepatitis B e antigen (HBeAg) status, and HBV DNA level into a predictive model for HCC risk. Validation was performed in Asian populations, specifically in hospital-based cohorts from Hong Kong and Korea, where approximately 82% of patients had cirrhosis at baseline. The score’s discrimination for HCC risk prediction was good in non-cirrhotic Asian patients, with a 5-year AUC of 0.783, but decreased to 0.698 in patients with cirrhosis. No robust validation has been performed in Western populations, and the medical literature notes that further validation is needed before applying REACH-B in non–Asian cohorts [80]. Additionally, the performance of the REACH-B score is greatly reduced in patients receiving antiviral treatment because HBV DNA is typically suppressed to undetectable levels, weakening a model that depends strongly on viral load. In a cohort of 1092 Asian patients treated with entecavir or tenofovir, the REACH B score showed poor discrimination, with a 5-year AUC of 0.572 [81]. A revised REACH-B (reREACH-B) model was recently developed by Kim et al. in 2024, incorporating platelet count into the original scoring system [82]. It was derived from 6949 Korean patients with chronic hepatitis B and externally validated in 7429 patients from Taiwan, Korea, and Hong Kong. The model demonstrated a nonlinear parabolic relationship between viral load and HCC risk, with the highest risk at approximately 6 log_10_ IU/mL and showed strong discrimination (0.844 in the derivation cohort and 0.813 in the validation cohort). As a newly developed model, further validation in diverse ethnic and geographic populations is needed [80,82].

The HCC Risk Estimating Score in CHB patients Under Entecavir (HCC-RESCUE), developed in 2017 in Korean patients with chronic hepatitis B receiving antiviral treatment, uses only three routinely available clinical variables: age, sex, and cirrhosis status. It demonstrated solid discrimination in the derivation cohort with a 5-year AUC of 0.81 and clearly separated patients into distinct three risk groups [83]. The score has been extensively validated in both Asian and non-Asian populations. Its simplicity, strong performance, and broad external validation make it particularly valuable in primary care environments and settings with limited resources, where more complex models may be impractical [76,83].

The CU (Chinese University)-HCC score, developed in 2010 in Hong Kong, incorporates age, albumin, bilirubin, HBV DNA levels, and cirrhosis [84]. It has been validated in both Asian and North American cohorts and offers good discrimination, particularly in non-Asian populations, with AUC values up to 0.91 [84,85]. However, the performance is reduced in patients with established cirrhosis and those receiving antiviral treatment [85]. Chon et al. reported that the CU-HCC score demonstrated an AUC of 0.81 after one year of antiviral therapy. The mean score declined markedly from 12.7 at baseline to 8.7 after one year, and this reduction remained stable over five years (8.4–8.8). These sustained improvements support the value of on-treatment risk reassessment, indicating that dynamic evaluation more accurately reflects the reduction in HCC risk achieved with antiviral therapy and enhances risk stratification for surveillance planning [86].

The GAG-HCC score (Guide with Age, Gender, HBV DNA, –HCC), developed in 2009 in a Hong Kong hospital-based cohort, incorporates age, sex, HBV DNA level, core promoter mutations, and cirrhosis. The GAG-HCC score demonstrated high predictive accuracy, with AUC values of 0.88 for 5-year and 0.89 for 10-year HCC risk prediction in the original cohort [87]. It was externally validated in both Asian and non-Asian populations, showing robust discrimination, though its performance declines in patients with established cirrhosis or those receiving antiviral treatment, similar to other HCC risk models [85,88].

Beyond the properties of each score, comparative studies provide additional insight by examining how these models perform across diverse populations and clinical contexts. In Asian patients receiving antiviral treatment, mPAGE-B demonstrated the highest discrimination, with 5-year AUROC values of 0.80–0.82, outperforming PAGE-B, CU-HCC, GAG-HCC, and REACH-B. HCC-RESCUE has shown promising discrimination in Asian patients on antiviral therapy, with AUROC values comparable to or higher than mPAGE-B and PAGE-B, but the evidence base is currently limited and further validation is needed [75,78,79,89]. In Caucasian patients on antiviral therapy, PAGE-B and mPAGE-B maintain robust performance, while Asian-derived scores (CU-HCC, GAG-HCC, REACH-B) perform less well. PAGE-B and mPAGE-B reliably identify low-risk groups with no HCC events over 3–5 years [76,77,90]. In both Asian and non-Asian untreated populations, the CU-HCC and GAG-HCC risk scores exhibit good to excellent discrimination, with CU-HCC ranging from 0.73 to 0.91 and GAG-HCC from 0.80 to 0.91 [85,91]. REACH-B is only suitable for non-cirrhotic, untreated Asian patients [82]. In patients with cirrhosis, the discriminatory performance of these scores declines, as cirrhosis itself becomes the dominant risk factor for HCC. However, CU-HCC and GAG-HCC may still show stronger performance in this population [85,91].

Other risk scores have demonstrated strong performance in derivation cohorts but have not yet undergone extensive external validation. The real-world risk score for hepatocellular carcinoma (RWS-HCC), developed in Singapore in 2020, incorporates age, sex, cirrhosis, diabetes, platelet count, and alpha-fetoprotein (AFP), all of which are routinely available clinical variables. The score enables clinicians to identify patients at low and high risk for HCC with high discrimination (AUC > 0.8) for HCC prediction, comparable to other AFP-inclusive models such as APA-B (which includes age, platelet count, and AFP) and REAL-B (which includes sex, age, alcohol use, diabetes, baseline cirrhosis, platelet count, and AFP) [76,88]. The hepatocellular carcinoma early surveillance score (HCC-ESC), which incorporates male sex, cirrhosis, and FIB-4, was developed in Hong Kong in 2020 and demonstrated promising discrimination in patients receiving antiviral treatment after HBeAg clearance. The AUCs for 5-year and 10-year HCC prediction were 0.771 and 0.790 in the derivation cohort, and 0.774 and 0.776 in the validation cohort, respectively [92]. Both RWS-HCC and HCC-ESC scores require external validation, particularly in non-Asian populations, to establish generalizability before routine clinical adoption.

#### 2.1.2. Hepatitis C Virus (HCV)

Fewer risk scoring systems exist for predicting hepatocellular carcinoma in patients with hepatitis C virus infection compared to those with hepatitis B virus infection because direct-acting antivirals for HCV have resulted in sustained virological response, which dramatically reduces HCC risk. The remaining at-risk HCV population is primarily those with advanced fibrosis or cirrhosis, a much smaller and more clinically diverse group, making large-scale derivation and validation of risk scores more challenging. FIB-4 is a simple tool for risk stratification in patients with HCV. Among individuals who achieve SVR in chronic HCV, a FIB-4 score ≥ 3.25 identifies patients at higher risk of HCC, with an annual post-SVR incidence of 1.22%, which exceeds the threshold for cost-effective surveillance. Conversely, non-cirrhotic patients with FIB-4 < 3.25 exhibit a substantially lower annual HCC incidence of 0.24% after SVR, suggesting that routine surveillance may be safely discontinued in individuals within this lower-risk category [93]. Beyond FIB-4, the Veterans Affairs (VA) HCC risk score was developed in the United States using a large cohort of more than 45,000 patients treated for chronic HCV within the VA healthcare system. The model incorporates age, platelet count, the AST/√ALT ratio, albumin, sex, race–ethnicity, HCV genotype, body mass index (BMI), hemoglobin, and AFP. These variables are summed to generate a linear predictor that is scaled from 0 to 100, with higher scores indicating greater HCC risk. A key finding from the derivation cohort was that four variables—age, platelet count, AST/√ALT ratio, and albumin—accounted for most of the model’s predictive power, with the remaining parameters contributing incrementally. The score demonstrates strong discrimination, achieving a C-statistic of 0.83 in both the derivation and internal validation cohorts, slightly outperforming FIB-4 alone (C-index 0.79). However, the absence of external validation limits its generalizability outside the VA population [94].

#### 2.1.3. Metabolic Dysfunction-Associated Steatotic Liver Disease (MASLD)

The MASLD-HCC score is a recently developed risk prediction model from Korean cohorts, designed to estimate HCC risk in patients with MASLD, including those without cirrhosis. The model incorporates five variables: age, sex, platelet count, and two cardiometabolic risk factors (CMRF 1 includes overweight or central obesity; CMRF 2 includes prediabetes or diabetes). Importantly, CMRF 1 and CMRF 2 are significant determinants of HCC risk irrespective of fibrotic burden or cirrhosis status (adjusted hazard ratios of 3.11 and 1.96, respectively). Even among patients with low fibrosis (FIB-4 < 2.67 or liver stiffness < 8 kPa), representing predominantly simple steatosis, those with CMRF 1 or CMRF 2 still developed HCC at annual incidence rates of 0.03–0.04%, exceeding the incidence reported in individuals without steatotic liver disease (0.003%). Across both the derivation and validation cohorts, the MASLD-HCC score demonstrated exceptional predictive performance, with a C-index of 0.84 in the derivation cohort, 0.83 in the internal validation cohort, and 0.93 in the external validation cohort. Moreover, it outperformed other leading HCC risk prediction models, including: aMAP (Age, Male, Albumin–Bilirubin, Platelets), ADRESS-HCC (Age, Diabetes, Race, Etiology, Sex, Severity of cirrhosis), THRI (Toronto Hepatocellular Carcinoma Risk Index), Agile 3+ and Agile 4+ score. Collectively, these findings indicate that the MASLD-HCC score not only delivers superior discrimination and calibration compared with existing models but also holds significant potential for improving precision surveillance, particularly among non-cirrhotic MASLD patients traditionally overlooked by conventional strategies [95].

**Table 1:** Etiology-specific risk scores.

Risk Score, Area/Country; 1st Author, Year	Cohort (Name/Derivation or Validation)	Number of Patients	Risk Score Parameters	Predictability, AUC/C-Index	HCC Risk	Risk Category and Incidence
Hepatitis B Virus (HBV)						
PAGE-B, Europe; Paratheodoridis, 2016 [31]	Derivation	1325	Age, sex, platelets	0.82 If addition of presence of cirrhosis: 0.84	5-year CI: 5.7%	5-year CI: ≤9 (low): 0% 10–17 (intermediate): 3% ≥18 (high): 17%
	Validation	490		0.81	5-year CI: 8.4%	5-year CI: ≤9 (low): 0% 10–17 (intermediate): 4% ≥18 (high): 16%
PAGE-B, Asia/Hong Kong; Yip, 2020 [75]	Validation	32,150		0.77	5-year CI: 4.8%	5-year CI: ≤9 (low): 0.5% 10–17 (intermediate): 4.4% ≥18 (high):14.0%
PAGE-B, United States; Kim, 2022 [76]	Validation	3101		0.73	0.75/100 person-years	5-year CI: ≤9 (low): 0% 10–17 (intermediate): 1.9% ≥18 (high):6.5%
mPAGE-B, Asia/Korea; Kim, 2018 [78]	Derivation	2001	Age, sex, platelets, albumin	0.82	5-year CI: 6.5%,	Annual incidence ≤8 (low): 0.2% within the first 4 years, 0.0% after 4 years of therapy 9–12 (intermediate): 1.1% within the first 4 years, 0.2% after 4 years of therapy ≥13 (high): 4.6% within the first 4 years, 1.0% after 4 years of therapy
	Validation	1000		0.82	5-year CI: 7.2%,	
mPAGE-B, Asia/Hong Kong; Yip, 2020 [75]	Validation	32,150		0.80	5-year CI: 4.8%	5-year CI: ≤8 (low): 0.5% 9–12 (intermediate): 3.3% ≥13 (high):14.2%
mPAGE-B, Australia; Jayawardena, 2025 [79]	Validation	1080		0.80	5-year CI: 1.9%	5-year CI: ≤9 (low): 0.44% 10–17 (intermediate): 1.0% ≥18 (high): 7.1%
REACH-B, Taiwan; Yang, 2011 [80]	Derivation (REVEAL-HBV cohort, Taiwan)	3584	Age, sex, ALT, HBeAg, HBV DNA	0.811 (3 years) 0.796 (5 years) 0.769 (10 years)	3.7% developed HCC within 12 years	A 17-point risk score was developed, with HCC risk ranging from 0.0% to 23.6% at 3 years, 0.0% to 47.4% at 5 years, and 0.0% to 81.6% at 10 years for patients with the lowest and highest HCC risk
	Validation (Hong Kong, Korea)	1505		0.902 (3 years) 0.783 (5 years) 0.806 (10 years)	7.4% developed HCC within 7 years	
REACH-B, Asia/Korea; Kim, 2017 [81]	Validation	1092		0.572	5-year CI: 4.8%, Patients with cirrhosis: 12.6%, Patients without cirrhosis: 0.7%	5-year CI: <8 (low): 8.8% ≥8 (high): 7.1%
reREACH-B, Taiwan, Korea, Hong Kong; Kim, 2024 [82]	Derivation	6949	Age, sex, ALT, platelet count, HBeAg, HBV DNA	0.844	0.63 per 100 person-years 10-year CI: 6.4%	Not reported
HCC-RESCUE, Korea; Sohn, 2017 [83]	Derivation	990	Age, sex, cirrhosis	0.768	1-year CI: 1.5%, 3-year CI: 5.0%, 5-year CI: 11.2%	5-year CI: ≤64 (low): 0.5% 65–84 (intermediate): 14.4% ≥85 (high): 37.1%
	Validation	1071		0.809	1-year CI: 0.9%, 3-year CI: 4.6%, 5-year CI: 8.9%	5-year CI: ≤64 (low): 2.1% 65–84 (intermediate): 9.3% ≥85 (high): 41.2%
HCC-RESCUE, United States; Kim, 2022 [76]	Validation	3101		0.77	0.75/100 person-years	5-year CI: ≤64 (low): 0.2% 65–84 (intermediate): 3.4% ≥85 (high): 6.9%
CU-HCC, Hong Kong; Wong, 2010 [84]	Derivation	1005	Age, albumin, bilirubin, HBV DNA, cirrhosis	0.85	10-year CI: 10.5%	5-year CI: <5 (low): 0.9% 5–19.5 (intermediate): 5.5% ≥20 (high):21.2%
	Validation	424		0.76 (5 years) 0.78 (10 years)	10-year CI: 10.6%	5-year CI: <5 (low): 1.7% 5–19.5 (intermediate): 9.5% ≥20 (high):21.1%
CU-HCC, North America; Abu-Amara, 2016 [85]	Validation	2105		0.85 (Asian) 0.91 (non-Asian)	5-year CI: 2.53%	5-year CI: <5(low): 0.49% 5–19.5 (intermediate): 7.3% ≥20 (high):8.5%
GAG-HCC, Hong Kong; Yuen, 2009 [87]	Derivation/Validation	820	Age, Sex, HBV DNA, core promoter mutations, cirrhosis	0.88 (5-year) 0.89 (10-year)	5-year CI: 4.4%, 10-year CI: 6.3%	Risk by group was not reported. <101 (low), ≥101 (high) Without core promoter mutations, <100 is defined as low risk at 5 years, and <82 is defined as low risk at 10 years NPV: 98.3–99%
GAG-HCC, North America; Abu-Amara, 2016 [85]	Validation	2105		0.87	5-year CI: 2.53%	5-year CI: <101 (low): 1.1% ≥101 (high): 9.8%
Hepatitis C virus (HCV)						
Veterans Affairs risk score, United States; Ioannou, 2018 [96]	Derivation	45,810	Age, platelet count, AST/√ALT, albumin, sex, race–ethnicity, HCV genotype, BMI, hemoglobin, AFP	0.70–0.77 (subgroup dependent)	2.8% at 2.52 years Subgroup: cirrhosis/no SVR subgroup (5.0 per 100 patient-years), cirrhosis/SVR (2.2 per 100 patient-years), no cirrhosis/no SVR (1.1 per 100 patient-years), no cirrhosis/SVR (0.3 per 100 patient-years).	The score is not a simple point-based system but rather a continuous risk estimate
Metabolic dysfunction-associated liver disease (MASLD)						
MASLD-HCC, Korea, Asia, Western countries; Chun, 2025 [95]	Derivation (Korea)	36,800	Age, sex, platelets, overweight/obese, or central obesity (CMRF1), prediabetes/diabetes (CMRF2)	0.84	0.2%, median follow-up: 5.1 years	Annual incidence <9 (low): 0–0.07% per year ≥9 (high): 0.21–2.01% per year NPV: 99%
	Internal Validation (Korea)	36,799		0.83	0.2%, median follow-up: 5.1 years	Annual incidence <9 (low): 0–0.05% per year ≥9 (high): 0.20–1.46% per year
	External validation (Asia, West)	4078		0.93	0.4%, median follow-up: 5.1 years	Annual incidence <9 (low): 0–0.04% per year ≥9 (high): 0.44–2.56% per year

Abbreviations: AUC, Area under the curve; C-index, Concordance index; CI, Cumulative incidence;; NPV, Negative predictive value; low, low risk; intermediate, intermediate risk; high, high risk; HBV, Hepatitis B virus; HCV, Hepatitis C virus; AFP, Alpha-fetoprotein; ALT, Alanine aminotransferase; AST, Aspartate aminotransferase; BMI, Body mass index; HBeAg, Hepatitis B e antigen; HBV DNA, Hepatitis B virus DNA level; CMRF1, Cardiometabolic risk factor 1 (overweight/obesity or central obesity); CMRF2, Cardiometabolic risk factor 2 (prediabetes or diabetes); MASLD, Metabolic dysfunction–associated steatotic liver disease; MASLD-HCC, Metabolic dysfunction–associated steatotic liver disease hepatocellular carcinoma score; PAGE-B, Platelet, Age, Gender score for hepatitis B; mPAGE-B, modified PAGE-B; REACH-B: Risk Estimation for Hepatocellular Carcinoma in Chronic Hepatitis B; reREACH-B, revised REACH-B; HCC-RESCUE, HCC Risk Estimating Score in CHB patients Under Entecavir; CU-HCC, Chinese University–HCC score; GAG-HCC, Guide with Age, Gender, HBV DNA and Cirrhosis for HCC score.

### 2.2. Mixed-Etiology Risk Scores (Table 2)

Recently developed risk scores, such as the aMAP score, THRI, and ADRESS-HCC, improve upon previously validated hepatitis B virus- and hepatitis C virus-related risk scores by extending applicability to non-viral etiologies and broader patient populations. Unlike earlier models (e.g., PAGE-B, REACH-B), which were originally developed for specific viral hepatitis populations and predominantly in Asian or Caucasian cohorts, these newer scores were derived and validated in large, multiethnic, international cohorts that included patients with non-viral liver diseases such as MASLD and ALD.

**Table 2:** Mixed-etiology risk scores.

Risk Score, Area/Country; 1st Author, Year	Cohort (Name/Derivation or Validation)	Number of Patients	Risk Score Parameters	Predictability, AUC/C-Index	HCC Risk	Risk Category and Incidence
aMAP, Asia/Europe; Fan, 2020 [97]	Search-B, Derivation (China)	3688	Age, sex, albumin–bilirubin, platelets	0.82	1-year CI: 0.4%, 3-year CI: 1.8% 5-year CI: 3.7% (95)	5-year CI: <50 (Low): 0.8% (95% CI: 0.3–1.3%) 50–59 (Medium): 4.2% (95% CI: 2.6–5.7%) 60–100 (High): 19.9% (95% CI: 12.8–26.5%) Cut-off value: 50 is associated with Sn: 86.5%, NPV: 99.5%
	9 cohorts Validation, (Asia/Europe)	13,686 9400 CHB 3566 HCV (2489 with cirrhosis and SVR), 720 NVH		0.82–0.87	3-year or 5-year CI: 1.3–7.0%	<50 (Low): 0–0.2% per year, 5-year CI: 0–0.8%; 50–59 (Medium): 0.4–1.0% per year, 5-year CI: 1.5–4.8% 60–100 (High): 1.6–4.0%per year, 5-year CI: 8.1–19.9% Cut-off value: 50 is associated with Sn: 85.7–100%, NPV: 99.3–100% Cut-off value: 60 is associated with Sn: 56.6–95.8%, PPV: 6.6–15.7%
aMAP, Japan; Johnson, 2022 [98]	Validation	3473		0.81 (95% CI: 0.79–0.82) Cirrhosis: 0.63 (95% CI: 0.60–0.67) Age ≥ 68: 0.73 (95% CI: 0.69, 0.763)	IR (per 1000 patient years): 10.9 (95% CI: 9.9–12.0)	IR (per 1000 patient years): <50 (Low): 0.98 50–59 (Medium):7.1 60–100 (High): 29.1
THRI, Canada; Sharma, 2018 [99]	Derivation	2079	Age, sex, etiology, platelets	0.77	10.8% if patients developed HCC during follow-up	5-year CI: <120 (Low): 1.2% 120–240 (Medium): 4.4% >240 (High): 15.4%
	Validation, from Rotterdam, Netherlands	1144		0.77	9.4% of patients developed HCC during follow-up	5-year CI: <120 (Low): 1.1% 120–240 (Medium): 4.9% >240 (High): 13.1%
ADRESS-HCC, United States; Flemming, 2014 [100]	Derivation	17,124	Age, diabetes, race, etiology of cirrhosis, sex, and severity of liver dysfunction	0.705	1-year CI: 5.6%	1-year CI: <2.15 (Low): 0% ≥4.67 (High): ≥1.5%
	Validation	17,808		0.691		
	Validation in HALT-C cohort	1005		Not reported	Not reported	1-year CI: 2.542 (group 1): 0.2% 4.228 (group 2): 1.0% 4.827 (group 3): 1.7% 5.794 (group 4): 4.6%

Abbreviations: AUC, Area under the curve; C-index, Concordance index; CI, Cumulative incidence; IR, Incidence rate; PPV, Positive predictive value; NPV, Negative predictive value; Sn, Sensitivity; low, low risk; medium, medium risk; high, high risk; aMAP, Age-Male-Albumin-Platelets score; THRI, Toronto HCC Risk Index; ADRESS-HCC, Age, Diabetes, Race, Etiology of cirrhosis, Sex, and Severity of liver dysfunction-Hepatocellular Carcinoma risk score; HALT-C, Hepatitis C Antiviral Long-Term Treatment Against Cirrhosis.

The aMAP (Age, Male, Albumin–Bilirubin, Platelets) score, first developed by Fan et al. in 2020, is a validated HCC risk model that incorporates age, sex, albumin–bilirubin (ALBI) score, and platelet count [97]. The aMAP score does not include virus-specific factors, underscoring its accessibility and applicability across different stages of clinical management. The derivation and validation cohorts included more than 17,000 patients from multiethnic populations—including Asian and Caucasian individuals—and from diverse etiologies such as chronic hepatitis B (cirrhotic and non-cirrhotic), hepatitis C (including post-SVR), and non-viral liver diseases. The score demonstrated excellent discrimination (C-index 0.82–0.87) and robust calibration for predicting HCC risk across both viral and non-viral etiologies. Risk categories are defined by cutoffs of 50 and 60. Patients in the low-risk group (aMAP < 50) had an extremely low 5-year cumulative incidence of HCC, with a negative predictive value of 99.5%. In contrast, individuals with an aMAP score > 60 fall into the high-risk category and therefore warrant continued HCC surveillance, with an estimated annual incidence of approximately 1.6–4.0% [97]. Although the score performs well across diverse ethnicities and etiologies, its discriminatory ability is attenuated in older individuals and in patients with cirrhosis, where competing risks of death and heterogeneous disease trajectories may diminish predictive accuracy [97]. In a Japanese cohort of 3479 patients with mixed etiologies, the aMAP score consistently stratified risk but showed a tendency to overestimate the 5-year probability of HCC (C-index 0.660–0.748)—a finding not observed in earlier validation studies. These results suggest that periodic recalibration of the aMAP score may be necessary to ensure its accuracy in contemporary real-world practice [98].

The Toronto Hepatocellular Carcinoma Risk Index (THRI), first introduced by Sharma et al. in 2018, was developed specifically for patients with cirrhosis [99]. Prior studies have shown that even among individuals with established cirrhosis, the long-term risk of HCC varies substantially across underlying etiologies. The THRI was designed to capture this etiology-specific variability using a unified risk model. The score incorporates four parameters—age, sex, etiology of cirrhosis, and platelet count—each assigned a weighted point value based on its hazard ratio for HCC development derived from multivariable Cox regression analysis. The total score stratifies patients into three risk groups: low risk (<120 points), intermediate risk (120–240 points), and high risk (>240 points). In the original study, the 5-year cumulative incidence of HCC was approximately 1.2% in the low-risk group, 4.4% in the intermediate-risk group, and 15.4% in the high-risk group, with strong performance in both the derivation and external validation cohorts (C-index 0.77) [99]. Despite these strengths, the THRI has notable limitations. The derivation cohort excluded non-cirrhotic patients, restricting its applicability to early-stage liver disease. Additionally, the original cohort lacked representation of Asian populations, among whom HCC epidemiology and competing risks differ substantially. Subsequent external validations in Asian and Swedish cohorts have demonstrated that although THRI can successfully stratify HCC risk, its discriminatory ability is lower than that observed in the original cohort, underscoring the need for population-specific refinement [101].

The ADRESS-Hepatocellular Carcinoma (ADRESS-HCC) score, first proposed by Flemming et al. in 2014, was developed in the United States using a large cohort of 34,932 liver transplant–listed patients with cirrhosis of various etiologies [100]. The model incorporates six clinical variables, age, diabetes, race, etiology of cirrhosis, sex, and severity of liver dysfunction, to generate a continuous estimate of an individual’s 1-year probability of developing HCC. In practice, higher ADRESS-HCC scores correspond to higher predicted 1-year HCC risk, although no universal cutoff values were defined in the original study. The model demonstrates acceptable discrimination, with C-index values of approximately 0.69–0.70. Because the derivation cohort consisted primarily of transplant-listed patients, most of whom had decompensated cirrhosis, external validation was performed using the HALT-C cohort. In this validation set, the lowest-risk group (score 2.542) corresponded to a 1-year HCC risk of 0.2%, with progressively higher scores predicting higher risk, up to 5.6% for the high-risk group (score 5.794). Several important limitations should be noted. First, the ADRESS-HCC model classifies cryptogenic cirrhosis within the nonalcoholic steatohepatitis (NASH) category, which may introduce misclassification bias as evidence indicates that cryptogenic cirrhosis is clinically and epidemiologically distinct from NASH, with differing metabolic characteristics, demographic profiles, and HCC incidence rates. Second, the model was designed solely to estimate 1-year cumulative HCC risk, and its performance over longer time horizons (e.g., 5-year or 10-year risk) remains unknown. Third, all derivation, internal validation, and external validation cohorts for the ADRESS-HCC model were derived exclusively from U.S. populations, limiting its generalizability to non-U.S. settings and to non-White ethnic groups [100].

In summary, aMAP has been validated in broader populations and provides more accurate, long-term HCC risk stratification across etiologies and ethnicities, while THRI is limited to cirrhosis and less reliable in Asian cohorts, and ADRESS-HCC is restricted to 1-year risk in cirrhosis with moderate performance [97,98,99].

## 3. Emerging Risk Scores Under Clinical Evaluation (Table 3)

Previous HCC risk scores have largely been static models relying solely on baseline variables. In 2023, Fan et al. advanced this paradigm by introducing the aMAP-2 and aMAP-2 Plus models, which incorporate longitudinal clinical data to dynamically track changes in aMAP scores and AFP levels, thereby capturing the temporal evolution of HCC risk [102]. In this stepwise framework, aMAP provides the initial baseline risk stratification, aMAP-2 enhances prediction by modeling dynamic biomarker trajectories, and aMAP-2 Plus achieves maximal risk enrichment through the integration of cfDNA fragmentomics. Collectively, these models represent a substantial improvement over static approaches and enable more individualized HCC surveillance. In the Search-B cohort, aMAP-2 demonstrated strong discriminative performance (AUC 0.83–0.84) and further subdivided baseline aMAP high-risk patients into groups with significantly different 5-year cumulative HCC incidences (23.4% vs. 4.1%, *p* = 0.0065). The aMAP-2 Plus model provided an additional layer of stratification, achieving AUCs of 0.85–0.89 and differentiating patients into high- and low-risk groups with annual HCC incidences of 12.5% and 0.8%, respectively (*p* < 0.0001). Despite their promise, the aMAP-2 and aMAP-2 Plus models have several important limitations. First, both scores were derived and validated exclusively in Chinese cohorts predominantly composed of patients with chronic hepatitis B, which may restrict generalizability to Western populations, non—Asian ethnicities, and other etiologies such as MASLD or HCV. Second, these models require serial measurements of biomarkers (aMAP and AFP), and aMAP-2 Plus additionally depends on advanced cell-free DNA fragmentomic profiling, which is not yet widely available or feasible in routine clinical practice. Third, prospective evidence demonstrating improved clinical outcomes, cost-effectiveness, or survival benefit when these models guide surveillance strategies is currently lacking [102].

**Table 3:** Emerging risk scores.

Risk Score, Area/Country; 1st Author, Year	Cohort (Name/Derivation or Validation)	Number of Patients	Risk Score Parameters	Predictability, AUC/C-Index	HCC Risk	Risk Category and Incidence
aMAP-2, China; Fan, 2023 [102]	Search-B, Derivation	3706	Longitudinal aMAP score, AFP	0.83	Not reported	5-year CI: Search-B: Low: 1.3% High: 14.2%, NPV: 98.2% aMAP defined high-risk and aMAP-2 low-risk: 4.1%, aMAP defined high-risk and aMAP-2 high risk: 23.4%
	Search-B, Validation	5796		0.84	Not reported	5-year CI: <0.46 (Low): 0.6% ≥0.46 (High): 7.2%
	PreCar, derivation and Validation	4226		0.73	Not reported	3-year CI: <0.46 (Low): 2.2% ≥0.46 (High): 11.1%
aMAP-2 plus, China; Fan, 2023 [102]	PreCar, derivation and validation	4226	Longitudinal aMAP score, AFP, cfDNA	0.85–0.89	Not reported	Bottom 90% (Low): 0.8% per year Top 10% (High): 12.5% per year

Abbreviations: AUC, Area under the curve; C-index, Concordance index; CI, Cumulative incidence; NPV, Negative predictive value; low, low risk; high, high risk; aMAP, Age-Male-Albumin-Platelets score; cfDNA, cell-free DNA.

## 4. Machine Learning Approaches to HCC Risk Prediction

The landscape of risk stratification is shifting from static clinical scores toward data-driven frameworks incorporating artificial intelligence (AI). In precision medicine, machine learning and deep learning approaches have shown strong performance in integrating high-dimensional clinical and molecular data for individualized risk prediction in various diseases. For example, deep learning models have been applied in ovarian cancer to identify drug-resistant subtypes and predict prognosis with high accuracy [103].

Building on these cross-disciplinary successes, recent advances in machine learning have enabled the development of highly accurate models specifically for HCC risk prediction. These include the PLAN-B model for hepatitis B virus infection, the SMART model for hepatitis C virus infection, and the logistic regression (LR) model for MASLD. The PLAN-B model, developed using a gradient boosting machine algorithm in chronic hepatitis B cohorts, incorporates cirrhosis status, age, platelet count, entecavir or tenofovir disoproxil fumarate use, sex, ALT, HBV DNA, albumin, bilirubin, and HBeAg status, and identifies a minimal risk group with an eight-year HCC risk of less than 0.5%. The PLAN-B model demonstrated significantly better discrimination than earlier models (PAGE-B, modified PAGE B, REACH B, and CU-HCC) in both the Korean validation cohort (C-index 0.79 compared with 0.64 to 0.74, all *p* < 0.05) and the Caucasian validation cohort (C-index 0.81 compared with 0.57 to 0.79, all *p* < 0.05 except modified PAGE B, *p* = 0.42) [90]. The SMART model from Japan, which analyzes post-SVR HCC occurrence in hepatitis C virus infection and was developed using a random survival forest approach, incorporates age, platelet count, AFP, gamma glutamyl transferase (GGT), albumin, AST, and BMI. Minami et al. demonstrated superior discriminative performance compared with conventional models (aMAP and ADRES), with higher C-index values in both the derivation cohort (0.936 compared with 0.762 or 0.679) and the validation cohort (0.839 compared with 0.830 or 0.807) [104]. For MASLD, the logistic regression (LR) model incorporates eight routinely available variables—age, educational level, height, weight, waist and hip circumference, and history of hypertension and diabetes—to predict HCC risk. Zhu et al. reported that the LR model achieved the highest accuracy (0.711 in the derivation cohort and 0.728 in the validation cohort) and strong discrimination (AUC 0.798 and 0.806, respectively). These findings suggest that models relying on routinely available demographic and clinical variables can support population-level risk assessment while maintaining feasibility and accessibility [105].

## 5. Discussion

### 5.1. The Importance of Risk-Based Precision Surveillance

Risk-based precision surveillance is essential because newly developed HCC risk scores now offer substantially superior discrimination than traditional indicators such as cirrhosis, liver stiffness, or FIB-4. Advanced models that incorporate multiple clinical predictors, longitudinal data, or machine learning, achieve C-indices approaching or exceeding 0.80 to 0.90, allowing much more accurate identification of individuals at risk. These tools overcome limitations of cirrhosis staging and liver stiffness measurement, which are prone to variability, operator dependence, and limited availability in routine practice. By directing surveillance toward patients with demonstrably elevated risk and reducing unnecessary imaging in low-risk groups, precision risk models enhance early detection, improve cost-effectiveness, and support a more equitable allocation of healthcare resources.

### 5.2. Remaining Limitations of Current Modalities and Scoring Systems

Optimizing HCC surveillance requires addressing the variable performance of abdominal ultrasound (US), the current standard modality. Although US is cost-effective and non-invasive, its sensitivity for early-stage HCC is limited and highly operator-dependent. Image quality is further reduced in patients with central obesity, hepatic steatosis, or chest wall deformities—conditions that are increasingly common with the rising prevalence of MASLD. In such sonographically difficult patients, uniform surveillance strategies may be inadequate. Risk stratification scores can help identify individuals who may benefit from alternative imaging modalities, such as abbreviated MRI or CT, to overcome the limitations of standard US.

Despite the rapid progress of scoring systems, several important limitations remain. First, many risk scores have not been developed or validated in certain racial or ethnic groups, including individuals from Africa or patients from the Middle East. Second, the potential risk of overfitting cannot be excluded, which may result in optimistic performance estimates and limited transportability even within similar populations. Furthermore, while most studies focus heavily on discrimination (e.g., AUC), data regarding the calibration of these risk scores—how closely the predicted risk matches the observed risk—are frequently lacking. As observed with the aMAP score in certain validation cohorts, models may overestimate risk in real-world settings [98], limiting their direct utility for defining strict surveillance thresholds. Accordingly, external validation and periodic recalibration are fundamental to confirm the robustness and clinical applicability of these scores. Third, prospective validation studies are still uncommon, and most newly developed risk scores show low positive predictive value, indicating weaker discrimination among patients in the highest risk categories. Fourth, many models rely on baseline variables and do not account for dynamic changes in liver disease activity or fibrosis regression over time, both of which can modify future HCC risk. Fifth, several scores include subjective clinical parameters (for example, cirrhosis in GAG-HCC, CU-HCC, RWS-HCC, and HCC-RESCUE) or depend on specialized biomarkers (such as HBV genomic mutations in the original GAG-HCC model or cell-free DNA in the aMAP-2 plus model), which may limit their feasibility in everyday practice. Finally, most current scores do not account for modifiable clinical factors, such as continued alcohol consumption or concomitant medications (e.g., aspirin, statins, sartans) [1]. Since these variables can significantly modify HCC risk or outcomes—as evidenced in post-ablation settings for sartans [106]—their omission limits the precision of risk estimation.

## 6. Conclusions

Advancing risk-based surveillance will require rigorous validation across diverse populations, a clear definition of surveillance thresholds, and practical frameworks to guide clinical adoption. With these advances, precision surveillance can transition from research to practice, enabling earlier HCC detection and more efficient use of healthcare resources.

## Data Availability

This study is a review article. No new data were generated or analyzed, and therefore data sharing is not applicable.
